# RBP4 Gene Variants Are Associated with Insulin Resistance in Women with Previous Gestational Diabetes

**DOI:** 10.1155/2014/269208

**Published:** 2014-02-09

**Authors:** Renata Saucedo, Arturo Zarate, Lourdes Basurto, Marcelino Hernandez, Edgardo Puello, Patricia Mendoza-Lorenzo, Patricia Ostrosky-Wegman

**Affiliations:** ^1^Endocrine Research Unit, National Medical Center, Mexican Social Security Institute, 06720 Mexico City, MX, Mexico; ^2^Hospital of Gynecology and Obstetrics, Medical Center La Raza, Mexican Social Security Institute, 02990 Mexico City, MX, Mexico; ^3^Department of Genomic Medicine and Environmental Toxicology, Instituto de Investigaciones Biomedicas, Autonomous National University of Mexico, 04510 Mexico City, MX, Mexico

## Abstract

*Objective*. This study aimed to examine possible genetic effects of some retinol binding protein-4 (RBP4) single nucleotide polymorphisms (SNPs) on the risk of gestational diabetes mellitus (GDM). In addition, the SNPs were examined for their possible association with insulin resistance at 6 weeks after delivery. *Methods*. This was a prospective study of 100 women with GDM and 100 participants with normal gestation who were evaluated at gestational week 30 and 6 weeks postpartum. Three SNPs of RBP4 (rs3758539, rs116736522, and rs34571439) were genotyped using TaqMan assay. The genotype distributions between GDM patients and normal controls were analyzed using logistic regression models. In addition, differences in clinical characteristics among subjects grouped by genotype were assessed using the analysis of covariance test. *Results*. The frequencies of the rare alleles were not significantly different between GDM patients and controls. However, we identified two variants rs3758539 and rs34571439 associated with insulin levels and insulin resistance in women with previous GDM. *Conclusion*. Noncoding SNPs of the RBP4 gene are not associated with GDM, but two SNPs showed associations with insulin resistance and insulin levels in women with prior GDM. Additional studies with increased sample size will be necessary in other GDM cohorts.

## 1. Introduction

Genetic studies of type 2 diabetes mellitus (T2D) suggest that it is a multigenic disease in which common variants in multiple genes interact with environmental factors to cause the disease [1–3]. Gene mapping has led to the identification of various chromosomal regions containing T2D susceptibility genes; one of them has been linked to an increased risk for type 2 diabetes in Caucasians and Mexican Americans and is located on chromosome 10q24 [4, 5]. The retinol binding protein-4 (RBP4) gene is located in this region and various studies showed that single nucleotide polymorphisms (SNPs) rs3758539, rs34571439, and rs116736522 in the RBP4 gene increased the risk of type 2 diabetes [6–11].

It is well known that women with gestational diabetes mellitus (GDM) are at a greater risk of developing T2D later in life. GDM shares several risk factors with T2D and shares similar pathophysiology [12]. Because of these striking parallels, recent work on the etiology of GDM has begun to evaluate the role of common variants in genes predisposing to T2D [13, 14].

The present study was directed to investigate some associations between the three SNPs of RBP4 previously shown to be present in persons showing some susceptibility to T2DM, during pregnancy and after delivery in women with GDM and healthy pregnant controls.

## 2. Materials and Methods

This is a prospective study conducted with 100 women with GDM and 100 pregnant healthy controls. Gestational diabetes was diagnosed by a 2 h 75 g oral glucose tolerance test at 24–28 weeks of gestation, the cutoff values being >5.2 mmol/L at fasting, >10.0 mmol/L at 1 h, and >7.8 mmol/L at 2 h. The protocol was approved by the hospital research ethics board and all participants gave written informed consent. Women with arterial hypertension, renal disease, liver disease, thyroid disorders, or other endocrine or chronic diseases were excluded. In the morning, at gestational week 30, anthropometric measurements of height and weight were obtained using a medical scale and blood samples were taken. A physical examination including measurement of blood pressure and a detailed history including family history of diabetes and obstetrical and medical information were obtained. After delivery, women with GDM and women with normal pregnancy returned to the clinical investigation unit for a 2 h 75 g OGTT at six weeks postpartum.

### 2.1. Laboratory Measurements

Venous blood samples for biochemical analysis were obtained in the morning after an overnight fast from an antecubital vein between 7:30 and 8:30 AM using vacuum tubes. The samples were centrifuged at 400 ×g for 15 minutes, and aliquots were obtained and stored at −70°C until assayed in a single run. Plasma glucose, triglycerides, and total cholesterol were measured by enzymatic assays with a Roche Cobas Mira analyzer using commercial kits (Stanbio Laboratory, Boerne, TX, USA). Insulin and RBP4 concentrations were determined by radioimmunoassay (RIA); insulin was measured using reagents from Siemens Healthcare Diagnostics (Los Angeles, CA, USA), sensitivity was 8.3 pmol/L and intra- and interassay coefficients of variation (CVs) were 5.2 and 7.3%, respectively. RBP4 was determined using reagents from Phoenix Pharmaceuticals (Belmont, CA, USA); sensitivity was 6.4 pg/mL and CVs were 4.9 and 8.3%.

Genomic DNA was isolated from anticoagulated blood by using the GFX Genomic Blood DNA Purification Kit (Amersham Biosciences) and stored at −70°C. Genotyping of selected SNPs in all study subjects was done using the TaqMan assay (Applied Biosystems) for the variants rs3758539, rs116736522, and rs34571439. The Taq-Man genotyping reaction was performed according to the manufacturer's protocol on an ABI Prism 7000 (Applied Biosystems, USA).

Body mass index (BMI) was calculated as weight in kilograms divided by the square of height in meters. The degree of insulin resistance was estimated by the homeostasis model assessment of insulin resistance (HOMA-IR) as follows: fasting glucose (mmol/L) × fasting insulin (mU/L)/22.5 [15].

### 2.2. Statistical Analyses

We examined Lewontin's *D*′ and the linkage disequilibrium coefficient *r* ^2^ between all pairs of biallelic loci. Haplotypes were inferred using the algorithm developed by the Broad Institute, Haploview. The allele distributions of polymorphisms among patients with GDM patients and normal subjects were evaluated by *χ* ^2^-test and Fisher exact tests accordingly, calculating odds ratios (ORs), 95% confidence intervals (CIs), and corresponding *P* values. We performed a multiple logistic regression analysis to estimate whether maternal age, maternal BMI, and RBP4 genotype were suitable as prognostic factors for GDM.

Because the frequency of the subgroups AA for SNP rs3758539, CC for SNP rs116736522, and CC for SNP rs34571439 is too low to perform statistical analysis, we combined these groups with the heterozygous group in a dominant model. Differences in clinical characteristics among subjects grouped by genotype were assessed using the analysis of covariance (ANCOVA) test, with age and BMI as covariates. Statistical analyses were carried out using Statistica version 8 (StatSoft, Tulsa, OK, USA). Significance was achieved at *P* < 0.05.

## 3. Results

The subjects' characteristics are shown in Table 1. GDM patients were significantly older and heavier than the control group. Of the biochemical parameters studied, total cholesterol and triglyceride levels were significantly higher in GDM patients.

The genotype and allele frequencies are shown in Table 2. All variants are in the Hardy-Weinberg equilibrium and during pregnancy, no significant difference was observed among the studied groups related to the polymorphisms. In the logistic regression analysis, the association between GDM and maternal age as well as weight was significant (odds ratio (OR) 1.21, 95% confidence interval (CI) 1.14–1.29, and *P* = 0.02; OR 1.04, 95% CI 1.01–1.09, and *P* = 0.03, resp.).

We estimated linkage disequilibrium among the three studied variants. As a result, rs3758539 and rs34571439 were in a tight LD block (*D*′ 0.9 and *r* ^2^ 0.6).

At 6 weeks after delivery in women with previous GDM, the insulin levels and HOMA-IR were significantly different between the G/G homozygotes and A allele carriers for rs3758539 and between A/A homozygotes and C allele carriers for rs34571439; the A allele carriers for rs3758539 and the C allele carriers for rs34571439 had a higher insulin and HOMA-IR. After adjustment for age and weight, the difference remained significant (Table 3).

In healthy controls at postpartum, cholesterol was significantly different between the G/G homozygotes and A allele carriers for rs3758539 and between G/G homozygotes and C allele carriers for rs116736522; the A carriers for rs3758539 and the C allele carriers for rs116736522 had a higher concentration. Moreover, RBP4 levels were significantly different between the G/G homozygotes and C allele carriers for rs116736522; the C allele carriers had a higher concentration. Further adjustment for age and weight abolished the difference observed between the two groups (Table 3).

## 4. Discussion

RBP4 has been proposed recently as an adipokine that may contribute to insulin resistance in muscle and liver, both in mice and humans [16, 17]. It is known that plasma RBP4 concentrations are positively related to insulin resistance, diabetes mellitus type 2, obesity, and a history of GDM [18–21]. However, discrepant results were also reported [22–25]. To our knowledge, this study is the first report of RBP4 polymorphisms in patients with previous gestational diabetes. Our data show that noncoding SNPs of the RBP4 gene are not associated with gestational diabetes. However, rs34571431 and rs3758539 showed associations with insulin levels and with insulin resistance in GDM subjects at postpartum. This association is consistent with the results obtained in T2D [6–11].

Our results vary from a previous study investigating RBP4 variants in Chinese women with GDM. In this study the variant rs3758539 was involved in the development of GDM [26]. By contrast, this variant was not found to be associated with GDM in Asian and Pacific Islander women [27].

The rs3758539 minor allele frequency observed for the GDM group was lower than those reported for Caucasian and Filipino with GDM. On the other hand, the frequency was similar to those reported for Chinese with GDM and higher than those reported for Pacific Islander [26, 27]. The variants rs34571431 and rs116736522 have not been evaluated in GDM.

The rs34571431 was in linkage disequilibrium with rs3758539. The rs34571431 is located at 5 bp downstream of the hepatocyte nuclear factor 1 motif, and it has been proposed that it could modify gene transcription efficiency by affecting the binding of transcription factors and RBP4 plasma levels. However, we did not find an effect of this polymorphism on serum RBP4 levels.

In addition, we found association between rs34571431 and rs3758539 with insulin concentrations and insulin resistance in GDM subjects at postpartum. The rare allele A carriers had higher insulin concentration and insulin resistance than G/G homozygote carriers. Previous studies reported that SNPs of RBP4 could increase diabetes susceptibility and decrease insulin secretion and insulin sensitivity. RBP4 is thought to affect insulin sensitivity by downregulating the activities of phosphoinositol-3-kinase and the phosphorylation of insulin in muscles. Furthermore, RBP4 can stimulate hepatic gluconeogenesis through stimulation of phosphoenolpyruvate carboxykinase. However, the mechanisms by which noncoding RBP4 variants could decrease insulin secretion are unclear.

This study is subject to certain limitations. We compared the genotype frequencies in GDM patients with those in unmatched controls. A control group consisting of age- and BMI-matched pregnant women without GDM may be more suitable for identification of the GDM susceptibility genes. Our study was underpowered to detect associations of RBP4 single nucleotide polymorphisms with GDM, probably because of their low frequencies, which may have resulted in some associations being overlooked. Using the single SNPs with the largest difference in minor allele frequency between cases and controls, we would need over 2350 cases and 2350 controls for 80% power to find a difference in rs34571439 or 7670 cases and 7670 controls to find a significant difference given the observed frequencies at rs116736522. We are aware that we have not corrected our statistical analyses for the number of comparisons made; the results therefore must be interpreted with caution. However, we did see an association of individual SNPs with quantitative traits related to glucose homeostasis in women with previous GDM, similar to the findings reported by Craig et al. who showed significant effects of RBP4 genetic variation on insulin resistance in Caucasians [7].

## 5. Conclusion

It seems that genetic variants rs3758539, rs116736522, and rs34571439 of the RBP4 gene are not associated with gestational diabetes, but the variants rs3758539 and rs34571439 show associations with insulin resistance and insulin levels at 6 weeks after delivery. This association is similar to that observed in T2D. Additional studies with increased sample size will be necessary in other GDM cohorts.

## Figures and Tables

**Table 1:** General characteristics of participants at gestational week 30.

	GDM	Control
	(*n* = 100)	(*n* = 100)
Age (yr)	32.8 ± 5.2*	25.9 ± 5.3
Weight (kg)	80.0 ± 11.3*	67.5 ± 15.2
Height (m)	1.55 ± 0.06	1.55 ± 0.08
BMI (kg/m^2^)	30.3 ± 5.0*	28.7 ± 7.5
Systolic blood pressure (mmHg)	108.4 ± 13.7*	104.8 ± 10.3
Diastolic blood pressure (mmHg)	72.0 ± 6.9*	66.5 ± 8.4
Fasting insulin (pmol/L)	64.8 ± 38.8*	52.1 ± 32.8
Fasting glucose (mmol/L)	5.36 ± 1.14*	3.9 ± 0.76
HOMA-IR	2.22 ± 0.3*	1.3 ± 0.16
Triglycerides (mmol/L)	3.85 ± 1.23*	2.78 ± 1.12
Total cholesterol (mmol/L)	7.21 ± 1.42*	6.92 ± 1.46
RBP-4 (*μ*g/mL)	4.92 ± 1.71	5.86 ± 1.64

Data are means ± SD.

*P < 0.05.

**Table 2:** *χ* ^2^ analysis of RBP4 polymorphisms among patients with GDM and healthy controls.

		GDM		Healthy controls				
Loci	Allele	*N*	Frequency	*N*	Frequency	OR (95% CI)	*χ* ^2^	*P*
rs3758539	G	93	0.93	91	0.91	1.39 (0.68–2.87)	0.83	0.36
	A	7	0.07	9	0.09			
rs116736522	G	97	0.97	96	0.96	1.07 (0.32–3.57)	0.01	0.91
	C	3	0.03	4	0.04			
rs34571439	A	91	0.91	88	0.88	1.44 (0.76–2.74)	1.28	0.26
	C	9	0.09	12	0.12			

**Table 3:** Association between SNPs and clinical features in two groups at postpartum.

	rs3758539				rs34571439				rs116736522			
	GDM		Healthy controls		GDM		Healthy controls		GDM		Healthy controls	
	G/G	A/G and A/A	G/G	A/G and A/A	A/A	A/C and C/C	A/A	A/C and C/C	G/G	G/C and C/C	G/G	G/C and C/C
BMI (kg/m^2^)	30.3 ± 3.9	29.0 ± 5.2	27.2 ± 11.0	23.1 ± 1.9	30.5 ± 3.9	28.6 ± 5.2	27.5 ± 11.6	23.8 ± 2.0	30.3 ± 4.1	27.6 ± 3.7	26.5 ± 10.7	28.3 ± 6.2
Glucose (mmol/L)	5.93 ± 1.9	6.5 ± 2.7	4.3 ± 0.5	4.2 ± 0.3	6.0 ± 1.9	6.1 ± 2.3	4.3 ± 0.05	4.2 ± 0.4	6.0 ± 2.05	5.9 ± 1.2	4.3 ± 0.5	4.1 ± 0.3
Insulin (pmol/L)	64.8 ± 80.4^∗†^	102.0 ± 77.4	36.6 ± 44.4	28.8 ± 7.2	64.2 ± 82.2^∗†^	98.4 ± 75.0	39.6 ± 46.2	22.8 ± 16.2	74.4 ± 84.0	55.2 ± 34.2	33.6 ± 40.2	60.6 ± 60.0
RBP4 (*μ*g/mL)	4.9 ± 1.0	4.4 ± 0.9	4.3 ± 1.0	4.6 ± 0.7	4.9 ± 1.0	4.4 ± 1.0	4.3 ± 1.0	4.4 ± 0.8	4.9 ± 1.0	4.2 ± 0.4	4.2 ± 0.9*	4.9 ± 0.3
HOMA-IR	2.7 ± 3.5^∗†^	5.8 ± 7.2	1.2 ± 1.6	0.9 ± 0.2	2.7 ± 3.5^∗†^	5.4 ± 7.0	1.3 ± 1.7	0.7 ± 0.5	3.5 ± 5.0	2.5 ± 1.5	1.1 ± 1.5	1.7 ± 1.6
Cholesterol (mmol/L)	6.1 ± 1.2	6.0 ± 1.5	5.5 ± 1.3*	6.4 ± 0.92	6.1 ± 1.2	5.9 ± 1.6	5.5 ± 1.3	6.0 ± 1.1	6.1 ± 1.3	5.3 ± 1.2	5.5 ± 1.3*	7.0 ± 1.1
Triglycerides (mmol/L)	2.3 ± 1.1	2.5 ± 1.2	1.8 ± 1.1	1.4 ± 0.3	2.2 ± 1.0	2.6 ± 1.1	1.9 ± 1.1	1.3 ± 0.5	2.3 ± 1.1	2.3 ± 1.2	1.7 ± 1.0	2.4 ± 1.3

Data are mean ± SD.

*P < 0.05.

^†^ *P* < 0.05 adjusted for age and BMI.
